# Advances in Microvascular Reconstruction of the Orbit and Beyond: Considerations and a Checklist for Decision-Making

**DOI:** 10.3390/jcm13102899

**Published:** 2024-05-14

**Authors:** Gian Battista Bottini, Veronika Joos, Christoph Steiner, Katharina Zeman-Kuhnert, Alexander Gaggl

**Affiliations:** 1 Department of Oral and Maxillofacial Surgery and Centre for Reconstructive Surgery, University Hospital of the Private Medical University Paracelsus, 5020 Salzburg, Austria; g.bottini@salk.at (G.B.B.); c.steiner@salk.at (C.S.); k.zeman-kuhnert@salk.at (K.Z.-K.); 2 Private Practice for Dentistry and Oral and Maxillofacial Surgery, 5026 Salzburg, Austria

**Keywords:** microvascular flaps, orbit reconstruction, free flaps, orbital exenteration, plastic and reconstructive surgery

## Abstract

This paper aims to discuss the microvascular reconstruction of the orbit and adjacent regions and to propose a checklist to aid the reconstructive surgeon in this challenging undertaking. The discussion is based on a literature review that includes 32 retrospective case series, 5 case reports published in the last 34 years in PubMed, and 3 textbook chapters. Additionally, it relies on the senior author’s expertise, described in a case series, and two case reports published elsewhere. Classifications and treatment algorithms on microvascular orbit reconstruction generally disregard patient-related factors. A more holistic approach can be advantageous: patient-related factors, such as age, comorbidities, prognosis, previous interventions, radiotherapy, and the wish for maximal dental rehabilitation and a prosthetic eye, have the same importance as defect-related considerations and can inform the choice of a reconstructive option. In this manuscript, we examine defect- and patient-related factors and new technologies, provide a checklist, and examine future directions. The checklist is intended as a tool to aid in the decision-making process when reconstructing the orbital region with microvascular flaps.

## 1. Introduction

Contemporary approaches to reconstructing the orbital region rely on microvascular free flaps. Previous articles have described various classifications and treatment algorithms to guide the reconstructive team in this difficult undertaking [1,2,3,4,5,6,7,8,9,10,11,12,13,14]. However, the above classifications have yet to reach global acceptance, likely because of their complexity, which limits their applicability in clinical practice [5,15,16].

This paper presents a discussion on the microvascular reconstruction of the orbit and adjacent regions and proposes a straightforward checklist for decision-making as a practical tool. The discussion is based on a literature review and the senior author’s expertise. 

## 2. Methods

A narrative review of the literature included papers published in PubMed in the last 34 years and contemporary textbooks on the topic. The database PubMed was searched using the words “orbit (orbital) reconstruction” and “microvascular flaps” and was limited to English. 

## 3. Results

The literature review yielded 32 case series, 5 case reports, and 3 textbook chapters on the orbital region’s microvascular reconstruction.

## 4. Discussion

The functional and aesthetic impact of defects involving the eye and the midface, along with their psychosocial burden, cannot be overstated [5,6,8,15]. The above mutilations mainly result from oncologic resections but occasionally from devastating traumatic injuries such as gunshot wounds, and their reconstruction is particularly challenging [14].

The reconstructive goals after orbital and midfacial resections recur across several publications [1,4,5]. These are a stable wound closure, a watertight separation between the neo-orbit, the nose, the brain, and the oral cavity, maintenance of a patent nasal airway, globe support (if the eye is preserved), intelligible speech, efficient mastication through adequate dental rehabilitation, and preservation of facial aesthetics [1,4,5].

Historically, split-skin grafts covered the bone after an orbital exenteration [17]. Obturators were the default technique used to obliterate maxillectomy defects [15,18]. However, obturators are plagued by several limitations. They cannot adequately address extensive defects due to their weight and insufficient oronasal seal, resulting in oronasal reflux and hypernasality [1,4]. Furthermore, they are high-maintenance and require patient commitment due to the need for meticulous daily hygiene and repeated adjustments as the contours of the resection change with time [1,4,18]. Their advantages are a much simpler surgical intervention, shorter operative time, and quick dental rehabilitation [13]. In the senior author’s practice, obturators have a place only for limited maxillectomies if flaps are not indicated or after a flap loss as a fallback option. 

Regional flaps such as the temporalis muscle flap or the temporoparietal fascial flap cannot fill extensive defects due to their small volume and short reach limited by the length of their vascular pedicle [3,13,15,17]. Eskander used the temporoparietal fascial flap to cover a custom-made orbital floor implant to prevent infection and extrusion [13].

Free tissue transfers were introduced in the 80s and have represented “a tremendous breakthrough in the ability to reconstruct defects in one stage without the limitations of reach and orientation of regional myocutaneous pedicled flaps” [5]. Free flaps have superseded previous techniques, such as skin grafts, obturators, and facial prosthetics, in most indications and for most patients [1,2,3,4,5,6,7,8,9,10,11,12,13,14]. Over the years, original solutions have been tried, and techniques have been refined to enhance aesthetics and functional outcomes. There is widespread agreement nowadays that free flaps transfers represent “state-of-the-art” techniques for reconstructing the orbit and adjacent regions. 

Several experts have detailed orbital and midfacial defects’ characteristics and advised on how best to address them. In 2000, Cordeiro e Santamaria compared the maxilla to a hexahedron, whose roof is the floor of the orbit supporting the globe, and its floor is the hard palate. Both levels were to be reconstructed, and a midface defect classification was introduced [2,3,4]. A type IIIb defect corresponded to an orbitomaxillectomy reconstructed with a three-skin-island rectus abdominis myocutaneous flap [2]. Type IV was a palate-sparing orbitomaxillectomy to be covered with a single-skin-island rectus abdominis myocutaneous flap [2]. These authors relied on a somewhat limited choice of reconstructive tools, i.e., mostly soft-tissue flaps such as the rectus abdominis myocutaneous or the radial forearm fasciocutaneous free flaps in conjunction with bone grafts for the orbital floor or the radial forearm osteocutaneous flap [2,3,4]. The osteocutaneous radial forearm free flap has been used by some authors to reconstruct midfacial defects [13,18] The authors of the present review do not use this flap due to its donor-site morbidity—such as pathologic bone fracture—and favor the medial condyle femur (MFC) bone free flap to reconstruct small bone defects like the orbital walls, inferior rim, and zygoma due to its low donor-site morbidity, as demonstrated previously [19,20]. If a skin paddle is also necessary, a saphenous artery perforator flap can be harvested with the bone to build a composite osteocutaneous MFC flap [20].

In 2001, Okay et al. identified three midface defect classes, from limited to extended [1]. Class II defects included resections of less than 50% of the palate and dental alveolus, and class III defects included resections of more than 50% of the palate and alveolus. Both classes—II and III—could be further characterized in the case of inferior orbital rim involvement, acquiring the letter “f” for orbital floor and “z” if the zygoma was resected [1]. The authors proposed a therapeutic flowchart, including obturators, local flaps, and free flaps according to the missing tissues [1]. They indicated composite bone free flaps, such as the iliac crest with internal oblique muscle, as a suitable option for class IIfz and IIIfz defects [1]. Implant-supported orbital prostheses were used to camouflage orbital exenterations [1].

In 2004, Yamamoto et al. stated that a microvascular reconstruction of the midface should aim to restore the three maxillary buttresses: the first vertical nasomaxillar, the second horizontal zygomaticomaxillar strut (including the inferior orbital margin and orbital floor), and the third vertical pterigomaxillar [6,7]. Three categories of resections corresponding to the above pillars were identified. Category II included orbitomaxillectomy and orbitozygomaticomaxillectomy, sparing the pterygomaxillary strut, and category III included the third pillar [6,7]. They observed infection, resorption, and fistula with implants and non-vascularized grafts, especially in conjunction with radiotherapy [6,7]. Therefore, they strived to use vascularized tissue transfers as much as possible [6]. Andrades and colleagues acknowledge vascularized bone flaps as the default solution for patients undergoing radiation due to their resistance to infection and exposure [18]. The authors of the present review have repeatedly observed exposure, infection, and fistula with implants in irradiated patients and share the same philosophy [20]. In Yamamoto’s patients, the horizontal zygomaticomaxillary strut—the inferior orbital rim and floor—was reconstructed with a vascularized costal rib combined with a rectus abdominis myocutaneous flap based on the deep inferior epigastric vascular system, where the eighth and ninth costal ribs were supplied through the connection between their intercostal vascular system and the deep epigastric [7]. Alternatively, they used a composite chimaera flap based on the thoracodorsal vessels and composed of a latissimus dorsi myocutaneous flap in combination with a scapula osteotomized in a V-shape, where the medial border replaced the inferior orbital contour and orbital floor, and the lateral scapular border replaced the pterygomaxillary buttress and the base of the V became the zygomatic prominence [7]. In category III defects, they used a chimaera flap made of latissimus dorsi, with a V-shaped scapula and rib [7]. In some cases, the aesthetical outcome of the orbital contour and zygoma was remarkable, achieving excellent symmetry and projection in Japanese patients with typically pronounced cheekbones [7].

One weakness of their reconstruction was the little bone stock available for dental rehabilitation, as observed by Eskander [13]. To overcome the limitation of a single bone flap, in selected patients, the senior author favors a composite double bone flap reconstruction to simultaneously address periorbital and zygomatic aesthetics, support for the globe (if preserved), and dental rehabilitation [20,21,22].

In 2006, Futran et al. provided a historical perspective on the surgical developments in this area. Still, they lamented the insufficient aesthetic quality of the reconstruction of the bony contours around the eye and of the zygoma in their patients, advocating a multi-disciplinary approach and combining free tissue transfer with ancillary techniques such as local flaps and maxillofacial prostheses to achieve the best possible outcome [5]. They offered a decision tree suggesting soft-tissue free flaps for “pure” orbital exenterations [5]. For extended exenteration involving the orbitozygomatic contour and orbital floor, they combined a soft-tissue free flap such as the myocutaneous rectus abdominis or the latissimus dorsi with calvaria or rib bone grafts or a composite bone free flap such as the subscapular system where the scapular tip can replace the orbital floor and rim [5,23]. 

Brown is considered one of the pioneers of head and neck reconstruction and was the first to describe the use of the DCIA with internal oblique muscle for immediate reconstruction after maxillectomy in 1996 [24]. He distinguished three types of maxillectomies: total (including orbital floor and palate), high or superstructure maxillectomy (including the orbital floor and inferior orbital rim but sparing the palate), and low or infrastructure maxillectomy (sparing the inferior orbital rim and removing the palate) [24]. For a total maxillectomy, the bone was placed vertically to replace the orbital rim and support the cheek and upper lip. The internal oblique muscle was folded in a dorsal direction in the oral cavity to build the neo-palate [8,24]. In orbitomaxillectomy, the internal oblique muscle was folded in the same fashion dorsally into the oral cavity and suspended cranially to the periorbital skin to obliterate the exenterated orbit. Brown showed that the bare muscle undergoes atrophy and epithelialization, providing an excellent oral and nasal cavity lining. At the same time, an orbital prosthesis was used to camouflage the exenteration [24]. In 2000, Brown devised a well-known classification system for maxillectomy defects entailing a vertical (class 1–4) and a horizontal dimension (a) [25]. Class III was a hemimaxillectomy including the orbital floor and sparing the orbit, and class IV an orbitomaxillectomy [25]. He later described the use of the DCIA with internal oblique muscle in a retrospective case series of 24 patients in 2002 [26]. He subsequently expanded his classification in 2010 with Shaw adding two further classes and changing to the Latin numerals: exenteration plus partial hemimaxillectomy sparing the palate and alveolus (class V) and nasomaxillary defects (class VI) [8]. Class III defects involved orbital floor resection with preservation of the orbital content [8]. For these defects, either a free fibula flap, a thoracodorsal angular artery (TDAA) scapular flap, or a deep circumflex iliac (DCIA) with internal oblique muscle flap were used to rebuild the maxilla and the inferior orbital rim, and the orbital floor was replaced by a titanium mesh [8].

For class IV defects (hemimaxillectomy including the palate and alveolus plus exenteration), either the DCIA with internal oblique muscle or the TDAA with latissimus dorsi muscle utilizing the muscle to fill the orbit, or exclusively myocutaneous flaps such as the latissimus dorsi or the rectus abdominis, in consideration of the poor prognosis of some patients in this category of defects, were employed [8]. Finally, bone was not considered a strict requirement for class V defects. Therefore, a radial forearm flap or an anterolateral thigh was employed in combination with an orbital prosthesis [8].

In 2009, Hanasono et al. classified orbital and midface defects into three categories: (1) orbital exenteration; (2) extended orbital exenteration, which entails the removal of one or more orbital walls; (3) orbital exenteration with maxillectomy [12]. Their reconstructive algorithm considered radiation therapy and the patient’s desire to have an orbital prosthesis in addition to the extent of the defect. It is the only one that acknowledges some patient-related factors in the decision-making process [12]. They used skin grafts in pure exenteration and camouflaged the orbital hollowing (“open cavity”) with an orbital prosthesis if no radiation was involved [12]. The same defect was reconstructed with a regional flap or a free flap in the case of radiotherapy (“closed cavity”, where the orbit is filled with soft tissue to the level of the orbital rim) [12]. For extended orbital exenteration, they used a regional flap or a free flap, and finally, for an orbitomaxillectomy, a free flap [12]. Hanasono and colleagues used no vascularized bone flaps but only myocutaneous rectus abdominis or fasciocutaneous anterolateral thigh in this series [12]. Subsequently, they published a large retrospective series of reconstructions of skull base resections, including orbitomaxillectomies [27]. Malignant tumors from the orbits can extend into the anterior cranial fossa, and tumors from the infratemporal and pterygopalatine fossae can invade the medial cranial fossa [27]. In the above situations, orbital extenterations or orbitomaxillectomies are sometimes necessary [27]. Bulky soft-tissue flaps like the anterolateral thigh or the rectus abdominis were used to obliterate the orbitomaxillectomies and the thin radial forearm free flap or the serratus anterior muscle free flap for orbital exenterations [27]. Patients who had previous surgery, irradiation, and chemotherapy received almost exclusively free flaps [27]. The same authors in 2013 reviewed an extensive series of midfacial resections and reconstructions. A total of 5 patients out of 246 underwent double flap reconstruction in the case of orbital maxillectomy using a fibula free flap for palatoalveolar reconstruction and the anterolateral thigh flap for external skin coverage or, in one patient, a combination of the fibula free flap for the alveolus and the composite serratus anterior muscle with vascularized rib for the orbital rim and the orbital floor [28]. Most of the orbital floor reconstructions, however, were conducted with titanium mesh bone grafts or polyethilene implants, the composite serratus anterior muscle with rib being used only if the patient had a history of prior irradiation and failed alloplastic reconstruction [28].

The authors noted that the patients for whom the eye was retained but the orbital floor was not reconstructed with a rigid layer developed orbital dystopia and enophthalmos [28]. Hanasono shifted from soft-tissue flaps to the free fibular flap for reconstructing the maxilla in highly functional patients with good prognoses because of the better lip support and the possibility of integrated implants for dental rehabilitation [28]. The free fibula flap was preferred to the DCIA or the scapula flap for its acceptable donor site morbidity, the possibility to perform simultaneous harvest and recipient site preparation, and its suitability for dental implants [28]. An osteocutaneous free fibula flap with a double-barrel configuration can reconstruct the maxillary alveolus, the palate, and the inferior orbital rim in a total maxillectomy [28,29]. It can provide support for orbital floor plates, recreate horizontal and vertical facial buttresses, restore the dental alveolus, allow for dental implant placement, and finally support the nose and the lips [29]. With the advent of surgical planning, Hanasono and colleagues have abandoned bone graft and titanium mesh and favor patient-specific titanium implants to achieve higher precision in the orbital floor and walls reconstruction [29]. 

In 2018, Urken—another pioneer of head and neck reconstruction—and Okay and colleagues revisited the Okay classification, introducing a class IV defect involving the maxilla, the inferior orbital rim, and the orbital floor, sparing the alveolus and palate [11]. Moreover, they included additional superscripts to specify further the vertical extension of the maxillectomy whereby the resection of the inferior orbital rim and orbital floor was described by the letter “f”, the removal of the zygomatic body with the letter “z”, an orbital exenteration with “o”, removal of facial skin with “s” and entry into the intracranial cavity with “ic”. For a class IV defect, they used either a locoregional flap, such as the galeal flap or the temporoparietal fascial flap, or free soft-tissue flaps such as the rectus abdominis, the radial forearm, and the pectoralis major myocutaneous free flaps [11]. Their choice for reconstructing the inferior orbital rim was a calvaria free bone graft covered by a temporoparietal fascial flap [11]. For their class II defect (a hemimaxillectomy with removal of the orbital floor), they used a DCIA flap as previously demonstrated by Brown [8,11,24]. Urken et al. used a titanium mesh for the orbital floor and a free bone graft for the malar prominence [11]. In disagreement with all other authors, they maintained that in cases of recurrent cancers or particularly aggressive neoplasms, such as mucosal melanoma or ameloblastoma, the guarded prognosis justified postponing a definitive reconstruction with free flaps for at least five years, leaving the cavity open and using an obturator until the secondary reconstruction was deemed safe from the oncologic perspective [11]. In agreement with all the other publications, a primary reconstruction is the default choice at the authors’ institution as it is technically more accessible and “averts significant psychological and emotional distress” for the patient compared to a secondary reconstruction [23]. In individual cases with bone erosion and infiltration of the skull base or the orbital apex, the treatment is planned in two stages. After completion of the tumor resection, samples from the specimen margins are taken for cryosection and intraoperative consultation with the pathologist. If the margins are clear, the cavity is tightly packed with an iodine gauze secured with stitches and, in the case of maxillectomy, with a plastic plate screwed to the adjacent bone. As soon as definitive histology is available, generally, two weeks later, a secondary reconstruction is performed if the margins are clear. Alternatively, a new resection with cryosection will precede the reconstruction. 

Uglesić and colleagues popularized the subscapular artery flap system, composed of the scapula, the latissimus dorsi, and the serratus anterior, using it for the primary reconstruction of orbitomaxillectomies in 27 patients [30]. These authors mainly harvested the bone flap on the angular vessel to increase the length of the pedicle, thereby achieving even more freedom for the placement of the various flap components. As in the DCIA with internal oblique, the muscle was epithelialized after three weeks and found to be better than using a myocutaneous flap as a neo-lining for the oral cavity [30]. Furthermore, the latissimus dorsi provided more bulk for the cheek than using the bone alone [30]. The main disadvantage of the subscapular artery system is the impossibility of a parallel two-team approach, prolonging the operation and increasing the anesthesiology risks for the patient [30]. Costa et al., in 2014, published their classification for maxilla reconstruction, where a palate-sparing (“superstructural”) orbitomaxillectomy is a type IV defect, a type III b is an orbitomaxillectomy with palate removal, and a type III b m includes a mandible resection. Differently from Brown, they preferred a horizontal orientation of the bone strut of the DCIA flap with the internal oblique muscle placed superiorly to fill the neo-antrum because they considered the periosteum to be a better lining for the oral cavity than the muscle [9]. They highlighted the advantages of bone flaps in providing an unyielding base for prosthetic rehabilitation in opposition to bulky myocutaneous flaps such as the rectus abdominis advocated by Cordeiro and colleagues [2,3,4]. Costa and colleagues used iliac or calvaria bone grafts to support the orbit [9]. Eskander also emphasized the importance of vascularized bone reconstruction as the foundation for achieving long-term stable midfacial contours, support for the globe, and dental rehabilitation [13]. In agreement with other publications, the authors of the present review concur that muscle free flaps are ideal for sealing compartments and filling big defects. However, they alone are not adequate for supporting the globe and do not allow dental rehabilitation or an aesthetic reconstruction of the orbital contours and zygomatic prominence [13,15,23].

Kesting et al. [10] published a classification of orbital exenteration and described four possible scenarios, from standard exenteration up to orbitomaxillectomy, providing a decision tree for addressing the various defects. Their recommended options were a split-thickness graft, a pedicled temporalis muscle flap, and a selection of free flaps like the radial forearm flap, the anterolateral thigh, the latissimus dorsi, or the scapular free flap [10]. Their type III defect was an orbital exenteration extended to the orbital roof with intracranial communication to be sealed with a galea periosteum graft, a fascia lata graft, a temporalis muscle flap, or a radial forearm flap [10]. At the authors’ institution, split-thickness grafts have no place to cover an orbital cavity. In agreement with Santamaria, the pedicled temporalis muscle flap is reserved as a fallback option for frail patients where prolonged anaesthesia poses an unacceptable risk [3]. The drawbacks of the pedicled temporal flap are the limited reach, the lack of volume, the impossibility of filling the orbit, and the unsightly temporal hollowing [10,13,17]. The median forehead can also cover an orbital defect even though the aesthetic outcome is modest due to the forehead scar and orbital hollowing [17].

The authors believe that apart from Hanasono’s classification, the remaining classifications and algorithms for reconstructing the orbital and adjacent regions lack applicability [1,2,3,4,5,6,7,8,9,10,11,12,13,14]. They all rely on retrospective analyses of small case series, thus representing low-level scientific evidence [31]. Finally, the most significant limitation is ignoring patient-related factors. The authors believe that from a practical perspective, every solution is valid if the patients can return to an adequate function, are satisfied with their appearance, and the complication rate is acceptable. The concept of the reconstructive ladder provides a file rouge for examining various options in orbital and midface reconstruction [15].

The simplest solution would be to leave the defect to heal by secondary intention packing the cavity with iodine gauze [17]. Healing requires several dressing changes and up to 10 weeks [17]. This technique has mainly historical interest. If the eyelids can be spared (provided oncological safety), the aesthetic advantage is enormous compared to a skin paddle from a distant region, as in a free flap [17].

Higher up the ladder, the choices become progressively more complex, ranging from partial- and full-thickness skin grafts to orofacial prosthetics, local flaps, and ultimately single or double free flaps, depending on the defect’s size and complexity, as well as the surgeon’s repertoire [12,21,32,33,34,35,36,37].

However, when choosing a suitable technique for solving a reconstructive problem, the surgeon should consider patient-related factors in addition to defect-related factors [15]. A more holistic approach can be advantageous; patient-related factors, such as age, comorbidities, prognosis, previous interventions, radiotherapy, and the wish for maximal dental rehabilitation and a prosthetic eye, have the same importance as defect-related considerations and can inform the choice of a reconstructive option. In the following text, the authors examine defect- and patient-related factors and new technologies, provide a checklist, and look at future directions. 

### 4.1. Defect-Related Factors

The surgeon should assess the size of the resection and the quality and functional requirements of the missing tissues. There is widespread agreement that as the resection becomes large, microvascular transplants are superior to obturators [4,5,26,34,38,39]. Free flaps can replace like with like, provide more positional flexibility, and allow for greater variation in size and shape compared to local flaps.

### 4.2. Choice of Flaps

Different types of free flaps can be utilized to reconstruct orbital defects, such as the following: radial fasciocutaneous forearm (RFF), fasciocutaneous anterolateral thigh (ALT), myofasciocutaneous latissimus dorsi, rectus abdominis muscle, serratus muscle, scapular flap, free fibula flap (FFF), deep circumflex iliac artery (DCIA) flap, and medial femur condylar flap (MFC) [40].

The above are popular choices in the head and neck reconstructive literature, except for the MFC flap. Nevertheless, the MFC flap is a workhorse in orthopedic reconstructive surgery. Initially introduced by Sakay et al. in 1991 for addressing non-union fractures of the upper limb, Kobayashi et al. applied it in 1994 for orbital reconstruction [41,42]. In Europe, Gaggl and Bürger popularized it in 2008 for head and neck and orthopedic indications [43,44,45]. Hamada et al. showed that the MFC flap, rich in osteoprogenitor cells through its vascularized periosteal cambium layer, has osteogenic capabilities, resulting in new bone production [46].

Hence, it proves beneficial for small bone defects in “hostile” recipient sites [47]. Moreover, it offers flexibility in both size and design while maintaining acceptable morbidity at the donor site [19,47,48]. Following its harvest, some discomfort in the knee may persist for up to six weeks. Nonetheless, the knee joint retains its stability and gradually restores its complete range of motion. Further, the scar position is aesthetically inconspicuous [19,48]. Sananpanich conducted an analysis of anatomical variations in the pedicles, the descending genicular artery, and the saphenous artery [49]. Gaggl, Rhee, Wong, and Banaszewski described the surgical technique for harvesting this free flap [43,48,50,51].

### 4.3. Type of Defects

According to Hanasono et al., orbital defects can be divided into three types: (1) orbital exenteration; (2) extended orbital exenteration (involving removal of one or more orbital walls); (3) orbital exenteration with maxillectomy [12].

The authors of the present paper consider orbital defects involving the zygomatic bone as extended exenterations. Finally, the authors identify a fourth type of defect, including patients with missing orbital walls, but where the eye and orbital soft tissues are preserved.

#### 4.3.1. Type 1. Orbital Exenteration

The primary functional requirement of a reconstruction is to establish a healed and stable seal between the neo-orbit and its adjacent compartments [5,10,23,34]. Obliteration of the neo-orbit avoids fistula formation and the spread of infection between the nose, the sinuses, the skin, and the brain [5,10]. The defect can be addressed using a soft-tissue flap like the RFFF, or ideally, a perforator flap such as the LAF, which offers greater thickness and reduced morbidity.

#### 4.3.2. Type 2. Extended Orbital Exenteration

When the resection involves the nasal cavity, sinuses, and skull base, establishing a watertight seal is essential to prevent fistula, cerebrospinal fluid leakage, and cerebritis [31]. Soft-tissue flaps like the LAF or the anterolateral thigh (ALT) may be viable options for these patients. Thicker soft-tissue flaps exhibit greater resistance to radiation compared to bone or thinner flaps, but they are unable to replicate three-dimensional structures [31]. Recreating the projection of the orbital margins and the zygomatic bone with a stable framework improves the aesthetics of the reconstruction. Cheek projection can be restored with alloplastic materials, bone grafts, or vascularized bone. However, the latter maintains its volume, whereas grafted bone is often subject to resorption. Following radiotherapy, grafts and implants are at risk of extrusion and contamination, particularly when the soft tissue envelope is fragile or compromised [21,38,52]. Therefore, for a more nature-like aesthetic outcome, one could opt for composite bone flaps like the MFC or the FFF, harvested with a suitable soft-tissue cuff or for a chimeric flap based on the subscapular artery system (SASF) [53].

#### 4.3.3. Type 3. Orbital Exenteration with Maxillectomy

**Figure 1:**
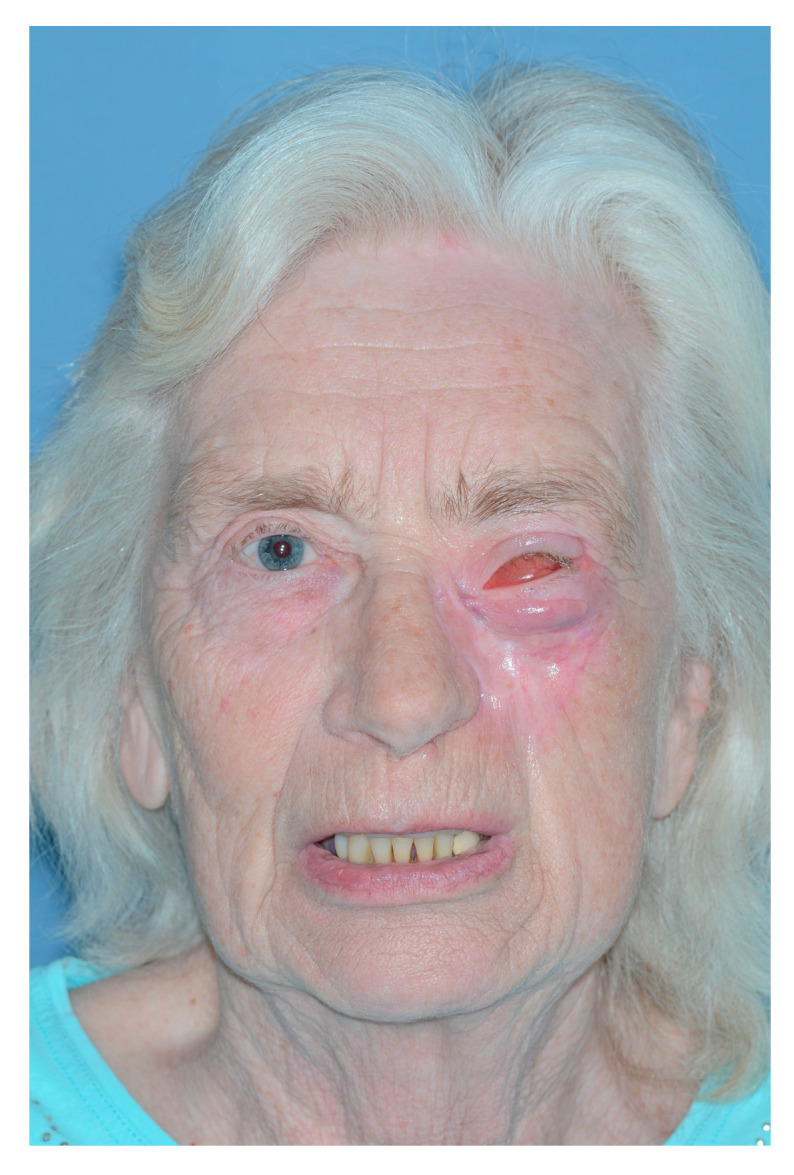
Patient at presentation after numerous resections and external beam radiotherapy for melanoma of the cheek on the left-hand side. At this stage, the melanoma was infiltrating the orbital floor, the orbital fat and the inferior oblique muscle, the maxilla’s frontal process, the lamina papiracea, and the anterior wall of the maxillary sinus. The eye was dislocated in a cranial position and rotated upwards.

**Figure 2:**
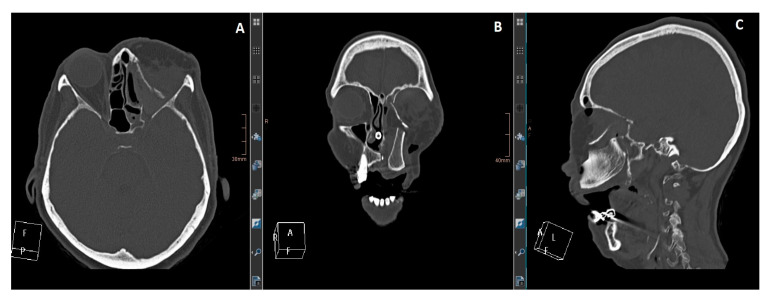
(**A**): Axial view. (**B**): coronal view. (**C**): sagittal view. CT imaging shows the maxilla reconstruction with a DCIA flap and the medial orbital wall and floor with an MFC flap. The reconstruction was delayed for two weeks after the resection to wait for definitive histology.

**Figure 3:**
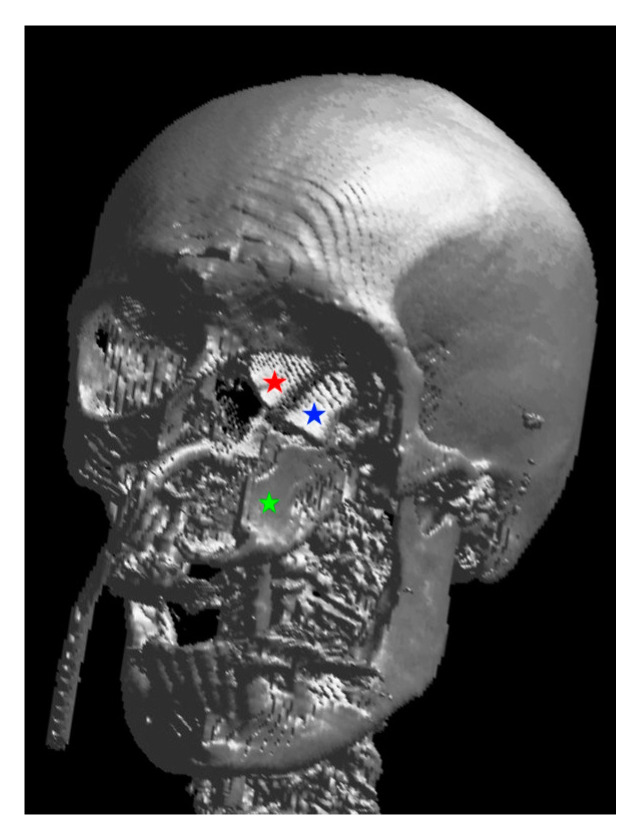
Three-dimensional rendering of the reconstruction: the red star marks the neo-medial wall, the blue star the neo-orbital floor, both belong to the MFC flap, and the green star marks the vertically oriented DCIA flap.

**Figure 4:**
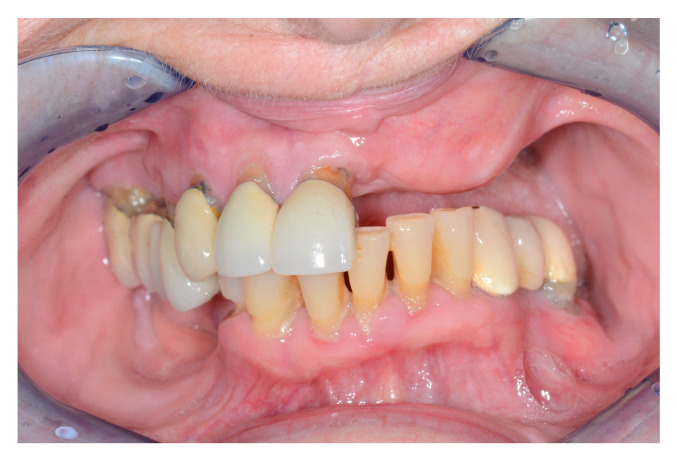
Intraoral view after alveolar ridge reconstruction.

**Figure 5:**
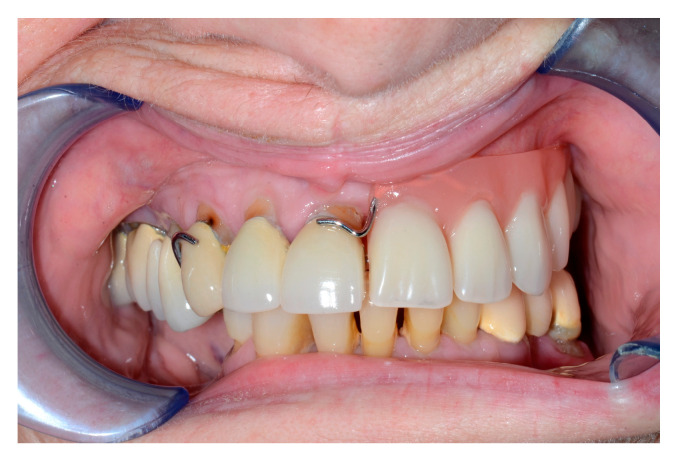
Intraoral view with denture.

**Figure 6:**
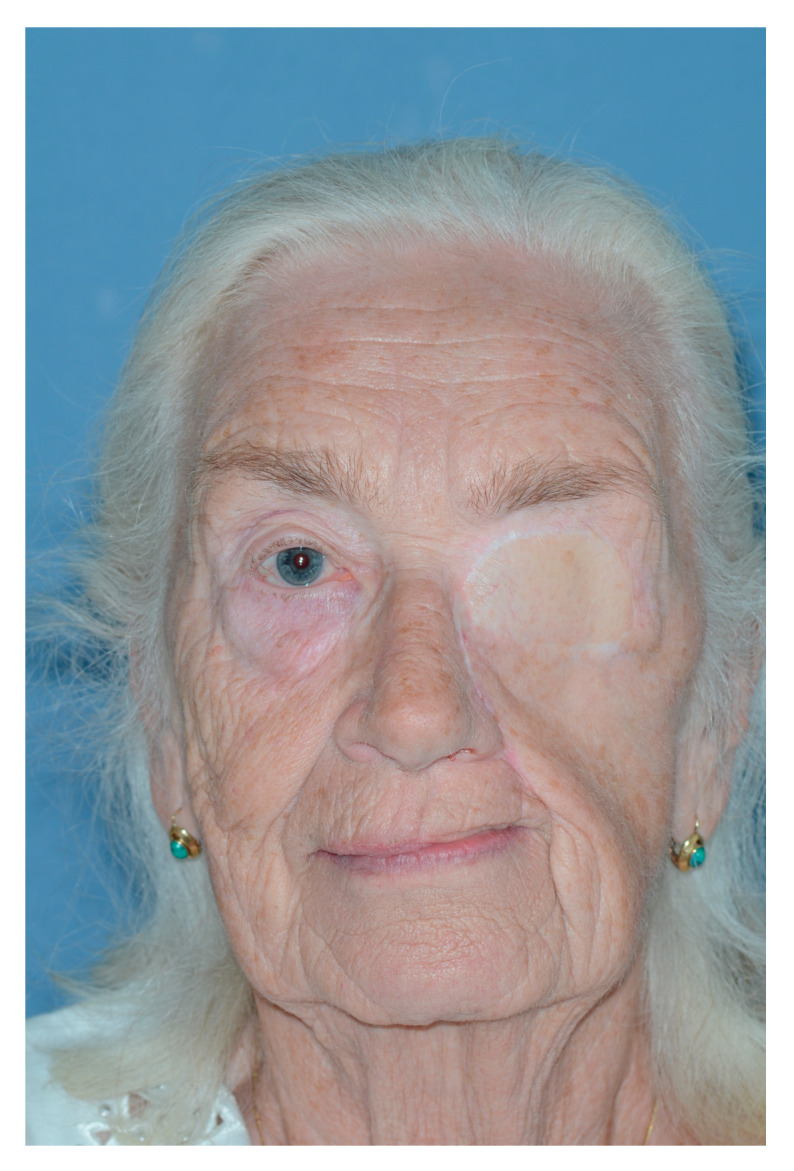
Outcome extraoral.

Here, support and volume are required to obtain an aesthetically pleasing and functional reconstruction. A DCIA with internal oblique muscle and skin in conjunction with an MFC (or a perforator flap) can satisfy the above requirements. The DCIA replaces the maxilla, while the MFC reconstructs the orbital walls, as seen in the patient below (Figure 1, Figure 2, Figure 3, Figure 4, Figure 5 and Figure 6). 

A water-tight closure between the neo-palate and the nose prevents hypernasality and the regurgitation of liquids and bolus and allows for efficient mastication, deglutition, and intelligible speech [14,18]. Restoring the above functions improves life quality for these patients [52,54]. Alternatively, soft-tissue flaps can obliterate these defects. However, muscular flaps may shrink and compromise facial aesthetics or hinder dental rehabilitation because of ptosis in the oral cavity [14,18]. Secondary corrections may be required to improve the outcome. On the other hand, the harvest of bone flap is more complex than soft-tissue flaps, especially concerning osteotomy design and an adequate three-dimensional reconstruction with an extended operation time. Additionally, the donor site morbidity of a bone flap can be problematic for the elderly patient, where early mobilization is highly desirable. Finally, bone flaps can function as rigid partitions to seal off separate compartments, provide support to nearby organs without yielding, integrate dental implants, or serve as a stable base for a denture, thereby enhancing oral rehabilitation [14]. Ultimately, patient-related factors should be prioritized when selecting a reconstruction after orbitomaxillectomy and will be examined further below.

#### 4.3.4. Type 4. Defect of One or More Orbital Walls with Preservation of the Globe and Orbital Soft Tissues

If the eye can be preserved, and one or more orbital walls must be removed, these partitions must be replaced with thin and rigid elements to support the globe and prevent enophthalmos, ocular dystopia and diplopia. Implants, bone grafts, and microvascular bone can fulfil the above needs. Implants are the most convenient option as they streamline and accelerate treatment while eliminating concerns about donor site morbidity [16]. However, if the recipient area has reduced vascularity or adjuvant radiotherapy is likely, microvascular bone is the safer option, given the higher risks of extrusion for implants [14,16]. The MFC flap is an excellent choice for reconstructing the orbital rims, the orbital walls, and the zygomatic bone. Finally, bone grafts are subject to resorption, non-union with the native bone, infection, and extrusion [14,44].

### 4.4. Choice of Recipient Vessels

The facial and temporal vessels are the default choice. The temporal arteries present several advantages: they have sufficient diameter for microvascular anastomosis, follow a reliable course, and are immediately adjacent to the orbit. Thus, the length of the vascular pedicle of the flap is not a concern [15]. One caveat: the temporal veins are thin-walled and require meticulous dissection to avoid perforations [55,56,57]. The facial vessels are also close to the orbit and the midface and offer adequate diameter for anastomosis. They can be exposed from the neck or even transorally to avoid an extraoral scar or if the flap pedicle’s length is insufficient, thereby forgoing an interpositional vein graft. However, an intraoral anastomosis is more challenging than an extraoral one due to the limited access and the reduced diameter of the facial vessels [58,59].

### 4.5. Patient-Related Factors

A history of radiotherapy, the likelihood of postoperative adjuvant radiotherapy, age, comorbidities, prognosis, and the wish for maximal dental rehabilitation and an orbital prosthesis must also be considered. In patients with a history of multiple interventions and radiotherapy, free flaps are favored over grafts or implants. This is because free flaps bring vital healthy tissues to an area that has been heavily scarred and poorly perfused due to previous treatments. Moreover, microvascular transplants can endure radiation much better than bone grafts in the case of postoperative radiotherapy [5,6,37].

In exenteration cases with maxillectomy, flap choice depends on the patient’s wish for maximal dental rehabilitation. To this end, bone flaps such as the FFF or the DCIA are well-suited for dental implants. However, the insertion of dental implants adds another layer of complexity to the overall treatment, implying additional procedures and potential complications. As such, they should be reserved exclusively for motivated patients with favorable prognoses. Poor dental hygiene and previous irradiation are contraindications for dental implants. The authors do not use zygomatic implants as they have a relatively high failure rate and can be associated with severe complications [47]. 

Prior irradiation, particularly of a non-vascularized bone graft, contraindicates implants for retaining a prosthetic orbit [5]. Finally, radiation-induced fibrosis restricts mouth opening, hindering the insertion of an obturator. This problem is exacerbated by monocular vision and declining manual dexterity associated with advanced age [39]. 

For such patients, the preferred approach is the obliteration of the orbit and palate using a soft-tissue free flap rather than relying on obturators that necessitate scrupulous hygiene and maintenance [5,15,31,39,40]. Similarly, advanced age, poor remaining monocular vision, and reduced manual skills contraindicate dental implants. The iliac crest or fibula free flaps entail higher donor site morbidity compared to soft-tissue flaps, potentially leading to gait disturbances that require extensive physiotherapy, abdominal wall weakness, and hip pain [60]. These options should be avoided in elderly patients who are not fit [60]. Additionally, peripheral vascular disease is a contraindication for harvesting a fibula free flap [60]. On the other hand, the donor vessels in the scapular system are typically free from atherosclerotic plaques, and the donor site facilitates early mobilization. Consequently, the scapular and parascapular flaps are adequate options for senior patients with peripheral vascular disease if bone reconstruction is necessary [53,61].

### 4.6. Orbital Prostheses

Some patients prefer to wear an eye patch, while others choose to have a prosthetic orbit. For the latter, the goal is to recreate an orbit that is symmetrical in shape and position to the contralateral side, thereby restoring facial harmony. A shallow concavity (“open cavity”) is ideal for receiving a prosthesis (possibly implant-retained). Alternatively, the orbital cavity should be filled (“closed cavity”) and present a slight convexity [12,34]. However, paradoxically, orbital prostheses can have a disconcerting effect on others due to the lack of eye movement and eyelid blinking, which are at odds with the ever-changing dynamic nature of facial expressions and the movements of the healthy contralateral eye. Eventually, some patients stop using their orbital prostheses due to this distracting effect, as well as the constant need for hygiene, regular maintenance, and the fear of accidental displacement [37]. By contrast, when the patient wears an eye patch, others’ attention is drawn to the contralateral functioning eye [5]. The clinician must inform the patients about the inherent limitations of a prosthetic solution to avoid nurturing unrealistic expectations, followed by disappointment and dissatisfaction [37]. Nevertheless, subjective perception of self does not coincide with objective aesthetic judgment; ultimately, the patient’s psychology matters most. 

### 4.7. Oncologic Safety and Timing of Reconstruction

It has been stated that an empty orbit lined by a skin graft, accessible for inspection, is safer from an oncologic perspective than a reconstructed neo-orbit filled with a free flap [35]. For the same reason, Urken et al. recommended a watch-and-wait policy before performing a delayed reconstruction for aggressive malignancies [11]. Pang et al. have suggested the adoption of a “delayed-immediate” protocol, which has the advantage of obtaining definitive histology of resection margins and allows time for designing bone flaps osteotomies, cutting guides, and pre-bending plates with the aid of computer-assisted design and computer-assisted modelling (CAD/CAM) technology [62]. Immediate reconstructions minimize the treatment burden and are preferable in cases with clear resection margins. Contemporary clinical follow-up is integrated by cross-sectional imaging and endoscopy, allowing for early detection of recurrence [5,24,26,31]. Life-long follow-up is advised, as recurrences are seen well beyond five years after resection [32]. There is no evidence to question the oncological safety of immediate reconstruction after maxillectomy in the literature [24,30,39,60].

In post-traumatic cases with extensive defects, early reconstruction with free flaps will prevent retractions and deformities that are extremely difficult to treat at a later stage [14].

### 4.8. Virtual Planning and Computer-Assisted Modelling

A precise reconstruction of complex shapes using bone flaps is difficult. Outcomes can improve with the aid of new technologies. Imaging obtained from computed tomography (CT) scans is used to create three-dimensional (3D) images of the skull and bone flap. Thanks to dedicated programs (computer-assisted design-CAD), the surgeon can simulate bone resections, flap osteotomies, and flap inset, thereby performing a virtual operation [29]. Milling machines or 3D printers use these 3D images to generate physical models, cutting guides for osteotomies and patient-specific implants (computer-assisted manufacturing-CAM). Conventional plates are bent precisely on the model before surgery and then sterilized, ready for use [38]. However, industry-made patient-specific implants and custom-made cutting guides are still costly. To limit the costs, virtual planning, model printing and pre-bending of conventional plates can be conducted in-house. Intraoperative CT provides imaging for assessing the bone reconstruction’s spatial arrangement in three dimensions with a lower radiation dosage than a fan-beam CT scanner [63]. This quality control allows for immediate corrections as required, avoiding returns to the operating room. Virtual surgical planning has dramatically improved precision and reduced operation times in orbitomaxillary reconstruction [29].

### 4.9. Double Simultaneous Free Flaps

Extended defects may require a double flap reconstruction. Each flap can be anastomosed separately to suitable receiving vessels in the neck or face. Alternatively, the two flaps can be linked sequentially in the vessel-depleted neck [64]. In the latter technique, the first flap is the “carrier flap” or flow-through flap, and the two flaps joined through the anastomoses form a fabricated sequential chimeric flap, which borrows its name from the Greek mythological creature made up from parts of different animals [65]. At the authors’ institution, Brandtner et al. joined the DCIA and an MFC flap in a chimeric flap to reconstruct the maxilla and the orbit [21]. De Cicco reported a case of orbitomaxillectomy reconstructed with a double composite flap (osteocutaneous fibula and DCIA with internal oblique muscle) where the fibula skin paddle underwent partial necrosis with suture breakdown and the reconstruction salvaged with a third free flap, a gracilis muscle anastomosed to the fibula in a sequential chimeric flap [22]. Bottini et al. described a retrospective case series on microvascular reconstruction of the orbit and adjacent regions where combinations of DCIA and MFC or DCIA with saphenous artery perforator flap were used for orbitomaxillectomies [20]. A double free flap reconstruction has the advantage of a superior outcome from the aesthetic (periorbital and zygomatic contours) and the functional perspective (globe support and dental rehabilitation) compared to a single flap. The downsides are a much higher challenge, prolonged operating time, which is risky for the patients and tiresome for the reconstructive team, and higher costs for the healthcare system. A straightforward checklist is provided below and intended as an aide mémoire in the decision-making process when planning how to reconstruct the orbital region with microvascular flaps (Table 1).

## 5. Conclusions

Free flaps are “state of the art” for reconstructing the orbit and adjacent regions. Vascularized bone reconstruction is foundational for achieving long-term stable midfacial contours, support for the globe, and dental rehabilitation. Classifications and treatment algorithms on microvascular orbit reconstruction disregard patient-related factors. However, a more holistic approach can inform the choice of a reconstructive option. A straightforward checklist is provided as an aide mémoire in the decision-making process when reconstructing the orbital region with microvascular flaps.

## 6. Future Directions

The first whole eye transplantation has already been performed; the difficulty is that the optic nerve is not able to regenerate; therefore, the patient cannot see through the transplanted eye [66]. Ongoing research efforts on nerve regeneration may overcome this obstacle in the future. In the future, tissue engineering could also replace the missing tissues with a scaffold, providing “mechanical stability and structural support for exogenous cell attachment and proliferation and facilitating the delivery of required growth factors for tissue regeneration” [67]. This would eliminate the need to harvest a flap and donor-site morbidity.

## Figures and Tables

**Table 1:** Checklist for decision-making when planning for the microvascular reconstruction of the orbital region.

**A. Patient-related factors:**
Multiple surgeries with persisting cancer: achieve clear resection margins before reconstruction (when in doubt, wait for final histology).
2. Previous irradiation and fistula: avoid implants, be prepared for multiple surgeries and flaps.
3. Old age or reduced fitness: favor soft tissue (perforator) flaps over bone flaps or obturators.
4. If the patient is frail, go for a local flap.
5. Young, fit, motivated patient with higher aesthetic expectations and wish for a prosthetic eye and dental rehabilitation: consider bone flaps and double flaps.
6. When adjuvant radiotherapy is likely, prefer bulky soft-tissue flaps to cover the bone framework.
7. Post-traumatic defects without infections: strive for early reconstruction to prevent retraction and deformity.
**B. Defect-related factors:**
Exenteration: prefer perforator free flaps over RFFF or local flaps.
2. Extended exenteration: consider composite bone flaps such as MFC, chimeric SASF, and FFF.
3. Exenteration with maxillectomy: consider SAFS, DCIA, or DCIA + MFC.
4. Resection of the orbital walls with eye preservation: favor MFC if soft tissues are compromised. Consider implants or grafts only if the local soft tissues are healthy.

RFFF = radial forearm free flap; SASF = subscapular artery system flap; FFF = fibula free flap; MFC = medial femoral condyle flap; DCIA = deep circumflex iliac artery flap.

## Data Availability

The data presented in this study are available on request from the corresponding author due to privacy restrictions.
